# The impact of COVID-19 on “biological aging”

**DOI:** 10.3389/fimmu.2024.1399676

**Published:** 2024-06-10

**Authors:** Fathima Humaira Amanullah, Tanvir Alam, Nady El Hajj, Yosra Bejaoui

**Affiliations:** ^1^ College of Health and Life Sciences, Hamad Bin Khalifa University, Qatar Foundation, Doha, Qatar; ^2^ College of Science and Engineering, Hamad Bin Khalifa University, Qatar Foundation, Doha, Qatar

**Keywords:** biological aging, epigenetic clocks, COVID-19, telomere attrition, DNA methylation

## Abstract

The global impact of the SARS-CoV-2 pandemic has been unprecedented, posing a significant public health challenge. Chronological age has been identified as a key determinant for severe outcomes associated with SARS-CoV-2 infection. Epigenetic age acceleration has previously been observed in various diseases including human immunodeficiency virus (HIV), Cytomegalovirus (CMV), cardiovascular diseases, and cancer. However, a comprehensive review of this topic is still missing in the field. In this review, we explore and summarize the research work focusing on biological aging markers, i.e., epigenetic age and telomere attrition in COVID-19 patients. From the reviewed articles, we identified a consistent pattern of epigenetic age dysregulation and shortened telomere length, revealing the impact of COVID-19 on epigenetic aging and telomere attrition.

## Introduction

The end of 2019 marked the outbreak of the severe acute respiratory syndrome coronavirus 2 (SARS-CoV-2) in China. The illness caused by the virus was named COVID-19 by the World Health Organization (WHO), which stands for “coronavirus disease 2019” (1). As of January 2024, the World Health Organization (WHO) has attributed over 774,075,242 confirmed cases and 7,012,986 deaths to COVID-19 (https://covid19.who.int/). SARS-CoV-2 is an encapsulated single-stranded RNA virus belonging to the genus Betacoronavirus (2). Individuals infected with SARS-CoV-2 may exhibit a range of symptoms from mild to severe (including fever, cough, difficulty breathing, sore throat, and loss of taste/smell) that manifest within two to 14 days of viral exposure (3). Even though COVID-19 can affect people of all ages, middle-aged and older adults have a higher hospitalization rate and risk of mortality when compared to children (2).

Aging is a time-dependent decline in physiological processes and integrity, manifested in the gradual loss of function and increased vulnerability to death. Although chronological age (cAge) is defined as the time elapsed from birth to a specific date, it does not fully reflect an individual’s physiological, physical, and mental functions (4). Therefore “Biological age” (bAge), which takes into account several factors including lifestyle, comorbidities, telomere attrition, and epigenetic alterations is a better measure of physiological or functional age (5). Measuring “bAge” is important for assessing the overall health of an individual and guiding towards healthy lifestyle habits. One potential biomarker for measuring bAge is DNA methylation (DNAm) whereby a methyl group is added to the 5th position of the cytosine ring to form 5-methylcytosine (6). Certain CpG sites in the genome show age-related changes in DNAm, which makes them particularly useful for developing age prediction biomarkers (7).

Recently, several “epigenetic clocks” have been developed to measure bAge utilizing regularized linear regression models trained on cAge and other physiological parameters of aging (8). In this regard, the first-generation clocks such as Horvath (comprised of 353 CpGs) and Hannum clocks (71 CpGs) were trained to predict cAge, whereas the second-generation clocks, such as PhenoAge (513 CpGs) and GrimAge (1113 CpGs) were trained to predict bAge as well as health outcomes such as likelihood of developing age-related conditions, including cardiovascular disease, diabetes, cancer and neurodegenerative diseases (8–12). The outcome of the epigenetic clocks further allowed measuring epigenetic age acceleration (EAA), which is calculated as the difference between cAge and the predicted age via epigenetic clocks. A positive divergence of bAge from cAge indicates EAA, whereas a negative deviation denotes epigenetic age deceleration (EAD) (13).

Studies have reported EAA in various diseases including cancer, cardiovascular disease, and aging-related disease (7, 14). Analysis of Berardinelli–Seip congenital lipodystrophy type 2 (CGL2), a segmental progeroid syndrome, revealed significant age acceleration in blood DNA of CGL2 patients using both first- and second-generation epigenetic clocks (15). Another study on individuals affected by Werner syndrome showed an increased epigenetic age of blood cells which is independent of changes in blood cell composition (16), this was not detected in Hutchinson-Gilford Progeria Syndrome (HGPS); a rare genetic disorder characterized by premature and accelerated aging beginning in childhood (17, 18). Similarly, EAA has been observed in patients suffering from infectious diseases such as Human Immunodeficiency Virus (HIV) (19) and Cytomegalovirus (CMV) (20).

Another well-studied marker of bAge is telomere length, which progressively decreases during biological aging resulting in chromosomal instability and loss of cell viability (21).

Telomeres are nucleoprotein structures that cap and protect the ends of chromosome arms (22). The cap-structure formed by telomeres maintains chromosome integrity and prevents chromosomal degradation. Telomere attrition during aging leads to senescence, apoptosis, or oncogenic transformation of somatic cells, hence affecting the health and lifespan of an individual. Telomere length measurement is used as a molecular biomarker for biological aging (23).

Several studies have investigated telomere biology in relation to premature aging disorders and found that telomere shortening is associated with diseases such as HGPS and Down’s Syndrome (24, 25).

**Figure 1:**
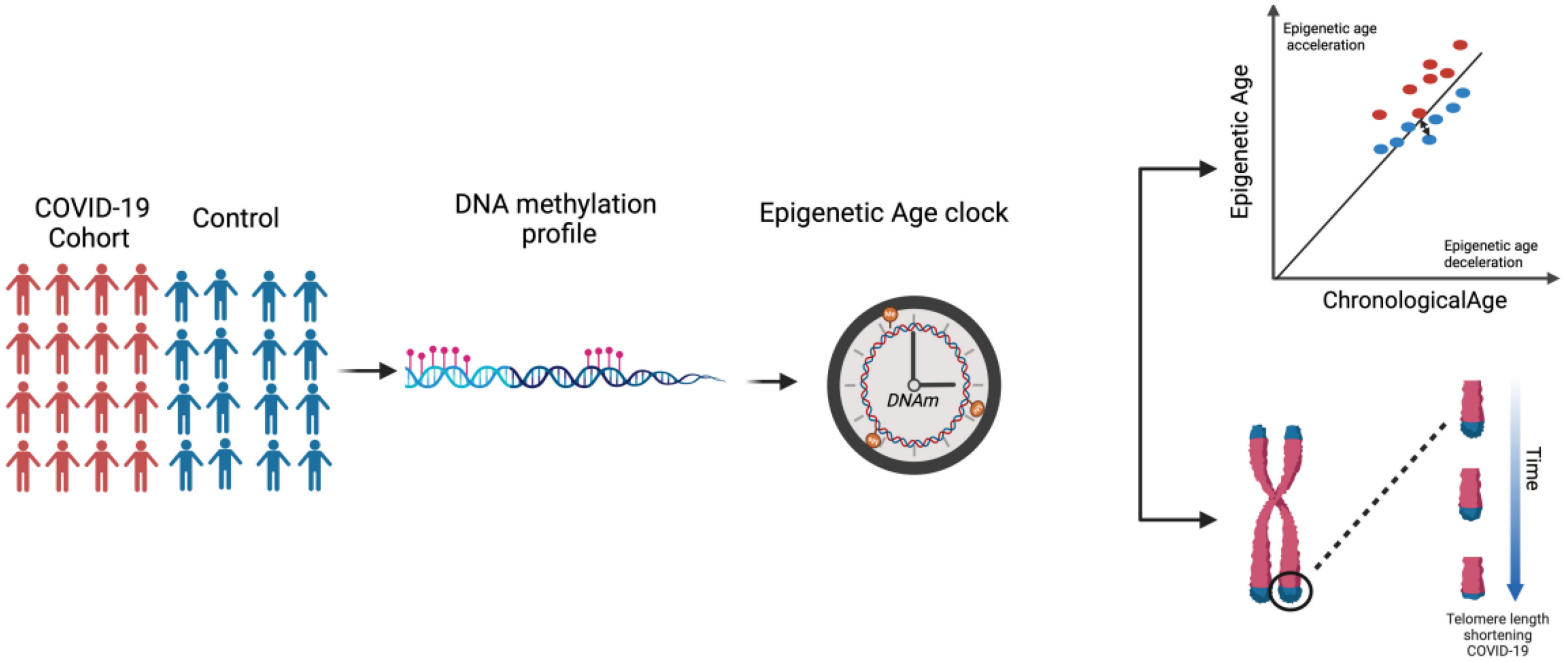
Schematic summary of the review concept.

Here, it is noteworthy to mention that both Epigenetic Dysregulation and Telomere Attrition are considered Hallmarks of Aging. As per our literature search, the extent of the association between COVID-19 and EAA has not yet been thoroughly investigated. While certain studies have reported an association (26), others failed to observe a difference in EAA (27). Hence, this review aims to explore the current landscape of research on biological aging in COVID-19 (**Figure 1**). We examined the evidence surrounding epigenetic age acceleration and telomere attrition in COVID-19 patients, understanding the multifaceted factors influencing susceptibility to severe outcomes and identifying potential avenues for future research.

## COVID-19 and chronological age

Several studies have investigated the association between age and COVID-19, and evidently, there is a strong effect of age on increased COVID-19 mortality (28). A single center retrospective cohort study performed on COVID-19 patients over 65 years found that patients older than 80 years exhibited higher mortality rates compared to the 65–80 year-old group (29). This can be explained by two main changes that occur in the immune system as we age. One is a decline in function known as immunosenescence which affects the ability to recognize and respond to pathogens. The other change is an increase in systemic inflammation called inflammaging. This happens because the alert system becomes overly active but less effective, at fighting off threats (30). cAge in humans is known to be associated with a deterioration of the body’s ability to protect itself against infections, due to the diminished effectiveness of the adaptive and innate immunity however this differs between individuals (31, 32). Older age has been associated with a weaker immune defense against pathogens and more comorbidities (33, 34). In addition, elderly patients have age-dependent defects in the function of B and T lymphocytes and a significant decline in humoral and cell-mediated immune functions. Cytokine and chemokine signaling is altered in elderly patients, with type 2 cytokine response preferred over type 1 response, which, in turn, impairs the cell-mediated immune response to infectious challenges. In addition, the increased production of type 2 cytokines may lead to poor outcomes, as it may weaken the control of viral replication and cause a prolonged proinflammatory response (35, 36).

Mortality is elevated among patients with pre-existing health conditions. Elderly patients with multiple comorbidities such as cardiovascular, neurological, respiratory, and metabolic diseases are at an increased risk of death from COVID-19 (37). A study investigating the population risk factors for COVID-19 mortality with bivariate and multivariate analyses found that patients with comorbid illnesses such as Chronic Obstructive Pulmonary Disease, Alzheimer’s disease, Asthma, and Lung Cancer exhibit high mortality rates suggesting that comorbid illnesses influence the mortality rate more than aging alone, which may be the reason why countries with a higher percentage of older people may witness more deaths from COVID-19 (38). Therefore, research on aging and COVID-19 indicates that for symptomatic cases, disease severity varies with age and other underlying health conditions. The association between cAge and the high mortality rate of COVID-19 in elderly patients may be attributed to health conditions, immunosenescence, or weak immune functions (39).

However, age may be an independent risk factor for COVID-19 severity or mortality, as shown in a population cohort study that examined the association between age and COVID-19 mortality. This study found that overall participants aged ≥75 years had a 13-fold greater mortality risk than those aged <65 years. In addition, this study revealed that participants older than 75 years had a 4-fold mortality risk when compared to the group of participants <65 years old and with the same risk factors as the older group (40). Hence, this study concluded that although comorbidities are a risk factor; age is an independent risk factor for COVID-19 severity or mortality. Several additional studies have also shown that the proportion of infections that progress to severe disease or death increases with age, especially in individuals above the age of 50 years (41–44). Similarly, when comparing disease severity and hospitalization among different age groups, it was observed that hospitalization rates and disease severity significantly increased with age. Pediatric patients with COVID-19 have a good prognosis, whereas adults with underlying conditions and the elderly have a higher mortality rate (45). Therefore, age may be a risk factor for the severity of COVID-19 (44).

## COVID-19 and biological aging (i.e. epigenetic age)

A study conducted by Mongelli et al. determined the bAge of 117 individuals who had recovered from COVID-19 (referred to as post-COVID-19) and 144 healthy participants using pyrosequencing focusing on CpG islands that have previously been identified as reliable indicators of bAge developed by Beckaert et al. The results indicate an increase in bAge among the post-COVID-19 group with an acceleration of DeltaAge by approximately 5.25 years, beyond the normal range (26, 46). These findings suggest that recovering from COVID-19 may lead to an accelerated aging process at the biological level. In an alternative study, on 407 COVID-19 samples, a higher Delta age (or as referred to by the authors as the Youth capital (YC)), which is the variance between an individual’s bAge and cAge, was consistently linked to reduced odds of severe symptoms when assessed using the Gonseth-Nusslé, Hannum, and PhenoAge clocks (47).

One study used five epigenetic clocks (PhenoAge, GrimAge, Horvath, Hannum, and Skin&Blood Clock) and a surrogate telomere length estimator to measure epigenetic age and telomere length attrition in three groups of patients: uninfected controls, non-severe COVID-19 patients, and severe patients. The calculated EAA showed a significant DNAm age acceleration across different clocks including Horvath, Hannum, PhenoAge, and GrimAge clocks in severe COVID-19 patients (48). Similarly, non-severe COVID-19 cases exhibited significant DNAm age acceleration in the Horvath, Hannum, skin&blood, and GrimAge clocks. Further analysis of epigenetic age dynamic acceleration across each COVID-19 disease phase revealed an acceleration from the initial phase, which was partly reversed in later phase. A similar study by our group, using the same epigenetic clocks and surrogate telomere length estimator, observed a significant EAA measured via the Hannum, PhenoAge, and GrimAge clocks in COVID-19 patients with acute respiratory distress syndrome (ARDS). Our study also observed EAA across several phases of the disease (49). Additionally, comparing DNAmAge in COVID-19 patients who died to those who recovered at both baseline and final follow-up revealed EAA only in the GrimAge clock. Interestingly, the Horvath, Hannum, and PhenoAge clocks showed a significant decrease in EAA at the last recorded time point before recovery. This suggests that EAD is associated with recovery from severe COVID-19 (49), which is consistent with a recent study by Poganik et al. reporting a significant reversal of biological age in COVID-19 affected females following discharge from the ICU using the PhenoAge and GrimAge clocks (50).

Additionally, a genome-wide study using the Illumina Infinium Methylation EPIC BeadChip850K (EPIC array) on 190 COVID-19 patients showed that epigenetic signatures at the time of hospital admission can significantly predict the risk of severe outcomes from COVID-19. By considering a 21CpG site signature, a logistic regression analysis was performed showing that the two groups, mild and severe, were distinguishable by 21 CpG epi-signatures. In addition, this study validated an association between epigenetic age acceleration and severe prognosis using the GrimAge clock. The results revealed a significant increase in EAA in severe COVID-19 cases compared to mild cases (51). In contrast to the aforementioned investigation, this study showed no consistent acceleration in epigenetic age compared to cAge in COVID-19 samples using Horvath, Skin&blood, Hannum Clock, and their recently described age-predictor for blood (27). This could be because epigenetic age changes occur as SARS-CoV-2 infection persists over time (26). Also, additional factors, such as the small sample size, the controls, and the patient samples not being matched by age and gender, may have affected the results. **Table 1** summarizes the studies focused on the assessment of bAge in COVID-19 patients.

**Table 1:** Comprehensive overview of studies assessing biological age in COVID-19 Patients.

Study aim	Sample size of Covid-19	Method	Study outcome	Reference
Determine a DeltaAge acceleration in COVID-19 survivors	117	Pyrosequencing of defined CpGs to measure biological aging using Bekaert’s algorithm (46)	bAge acceleration in COVID-19 survivors	(26)
Evaluate epigenetic age acceleration in severe COVID-19 infections that require hospitalization	47	Targeted bisulfite amplicon sequencing of 3 age-associated region (*FHL2,CCDC102B, PDE4C)*	No evidence of accelerated bAge in severe COVID-19 patients	(27)
Estimate the epigenetic age of COVID-19 patients using epigenetic clocks	407	EPIC array	EEA in the COVID-19 patients using Horvath, Hannum, skinHorvath and GrimAge clocks compared to healthy controls.	(48)
Analyze the epigenetic landscape of immune cells during severe SARS-CoV-2 infection	9	EPIC array	Severe COVID-19 is associated with increased DNAm age and mortality risk according to GrimAge clock.	(52)
Assess the causal relationship between aging and COVID-19	34710	Mendelian Randomization	No causal relationship between epigenetic age and COVID-10 susceptibility	(53)
Assess the association between different measures of epigenetic age and COVID-19 severity	509	EPIC array	Higher YC using the Gonseth-Nusslé, Hannum and PhenoAge measures was associated with reduced odds of severe symptoms	(47)
Examine epigenetic age acceleration in COVID-19 patients with ARDS	87	EPIC array	Severe COVID-19 is associated with a significant increase in bAge using Hannum, PhenoAge and GrimAge.	(49)
Identify epigenetic biomarkers that could predict the clinical prognosis of patients	190	EPIC array	Significant EAA between the COVID-19 severe and mild groups using the GrimAge clock	(51)

The lack of reproducibility in these studies can be attributed to varying sample severity categorization and differences in comorbidities in the affected cohorts and controls. As there is no uniform standard to classify COVID-19, some studies define severity based on patient hospitalization, oxygen therapy, mechanical ventilation (26, 47, 48, 51, 52), or deceased status while other studies rely on the WHO clinical progression or Charlson severity index (47, 49). In addition, data stratification between genders can affect the results obtained from the epigenetic clocks, since it was shown that males are at high risk for severe disease and mortality by COVID-19 (54). Ethnicity can also influence the outcome and the severity of the disease and should be taken into consideration when performing such studies (55, 56).

Furthermore, the majority of epigenetic clocks exhibited variability and conflicting results across the different studies, however a notable consistency of increased bAge measured via the GrimAge clock was evident across numerous studies. This may be attributed to the fact that GrimAge was trained on factors closely related to the risk of respiratory diseases such as mortality and smoking, which may explain its effectiveness as an epigenetic marker for aging, particularly in the context of respiratory diseases (48, 49, 51, 52).

## COVID-19 and telomere length

Multiple studies reported an association between severe COVID-19 infection and shorter telomeres. Telomere shortening results from the incomplete synthesis of the lagging strand during DNA replication due to the inability of DNA polymerase to completely replicate the ends of chromosomal DNA usually as a consequence of either oxidative stress or inflammation (57, 58). In a prospective study, telomere length in hospitalized COVID-19 patients revealed a significantly higher proportion of COVID-19 patients with shorter telomeres when compared to the control cohort. Telomere attrition was associated with a higher risk of critical disease, defined as admission to the intensive care unit (ICU) or death without ICU (59). In another study on COVID-19 survivors, significant telomere shortening was observed following absolute human telomere length measurement (26). A similar finding was reported by Sanchez-Vazquez et al. where telomeres in severe COVID-19 cases were observed to be shorter than those in patients with mild COVID-19 symptoms (53, 60). Furthermore, a decrease in age-adjusted leukocyte telomere length was associated with 1.35 higher odds of fibrotic-like patterns four months after hospitalization. Hence, longer telomere length may be protective against post-COVID lung fibrosis, and shorter telomere length may lead to more severe pathologies due to the impaired regenerative abilities of cells post-SARS-CoV-2 infection (61). Telomerase enzymes can elongate shortened telomeres; hence, telomerase activation-based therapies can be used to improve the complications of severe COVID-19 however, further investigation is needed to confirm the safety of this therapy. (62). It was also shown that leukocyte relative telomere length measured in patients at two different time points (at admission and one year after discharge), revealed significant telomere shortening associated with fibrotic pulmonary sequelae (63).

DNAmTL measurements revealed telomere attrition acceleration in deceased COVID-19 patients between inclusion and end of follow-up and a significant change in dynamic telomere attrition acceleration when comparing patients who recovered versus those who died (49). It was in line with another study where they showed that individuals with severe COVID-19 displayed significant DNAmTL attrition acceleration compared to individuals with non-severe COVID-19 (48).

A recent study with 89 patients, including 61 females, and 28 males, observed that telomere length is consistently longer in females than in men across all age ranges (60). This is consistent with other findings reporting female COVID-19 patients to have a lower mortality rate than male patients (54, 64, 65). Hence, shorter telomere length may be fatal following SARS-CoV-2 infection since infections in individuals with shorter lymphocyte telomere length are more severe and patients associated with lymphopenia (62, 66). However, a few studies (49, 52) presented no evidence of significant telomere shortening in severe COVID-19 cases. For example, fluorescence *in-situ* hybridization (Flow-FISH) analysis performed on COVID-19 lymphocytes did not reveal significantly accelerated telomere attrition in the studied patients (27). In **Table 2**, we provide a summary of the studies assessing telomere length in COVID-19 Patients.

**Table 2:** Comprehensive overview of studies assessing telomere length in COVID-19 Patients.

Study aim	Sample size	Method	Study outcome	Reference
Determine changes in the epigenetic landscape of immune cells during severe COVID-19	9	DNAm telomere length estimator (140 CpG sites)	No significant telomere shortening in severe cases	(52)
Identify independent risk factors for the development of post-COVID fibrosis	76	Quantitative PCR assay(qPCR)	Telomere length is an independent risk factor for the development of fibrotic-like abnormalities	(61)
Investigate for telomere length alteration in COVID-19 survivors	117	qPCR Assay	Significant telomere shortening in post-COVID cohort	(26)
Evaluate if accelerated epigenetic age increases susceptibility to severe COVID-19	19	Flow-FISH	No significantly accelerated telomere attrition in severe cases	(27)
Assess if shorter telomere length is correlated with more severe COVID-19 pathology	89	qPCR	Shorter telomeres are associated with greater severity of COVID-19	(60)
Determine if shorter TL is associated with poor COVID-19 outcome	70	Flow-FISH	Telomere shortening is associated with a higher risk of ICU admission or death	(59)
Assess the causal relationship between aging and COVID-19	34,710	Mendelian Randomization	Severe COVID-19 causes telomere length attrition	(53)
Examine the impact of COVID-19 on Telomere length	87	DNAm telomere length estimator (140 CpG sites)	Telomere attrition acceleration in deceased patients but not in severe patients	(49)
Analyze the alteration in telomere length in COVID-19 patients and association with fibrotic sequelae	19	qPCR	Identified peripheral blood leukocyte telomere attrition in COVID-19 patients one year after infection	(63)

Although different methods and sample sizes were used to measure telomere length, we can observe a consistency across most studies indicating telomere length shortening. Therefore, despite the differences in methods and approaches used, a true biological effect is captured, confirming that telomere length can be used as a possible biomarker for COVID-19 outcome and severity.

## Conclusion

In conclusion, this review highlighted the impact of COVID-19 on biological aging and telomere attrition. The review focuses on how SARS-CoV-2 infection has been reported to perturb epigenetic age and telomere length. Multiple studies utilizing different epigenetic clocks unveiled epigenetic age acceleration and telomere shortening in COVID-19 patients, particularly in severe cases. However, there are limitations to the existing research, such as the usage of methylation data from whole blood to estimate epigenetic age. Most studies focused on mild and severe patient cohorts, additionally, the lack of standardized severity categorization and unspecified severity levels poses challenges for comparison and analysis. To address these limitations, future studies should explore epigenetic age analysis in alternate tissues to validate the previous findings. Furthermore, standardizing the severity classification according to the WHO clinical progress scale could enhance comparability among studies. As research in this field progresses, more studies are required to assess the value of epigenetic clocks as biomarkers or predictors of COVID-19 disease severity, ultimately advancing our ability in early disease management. By addressing these challenges and expanding our knowledge in this field, we can better prepare for future pandemics and improve overall public health outcomes.

## Author contributions

FH: Writing – original draft, Writing – review & editing. TA: Writing – review & editing. NE: Supervision, Writing – review & editing. YB: Writing – original draft, Writing – review & editing.
